# Supplementary material for: High-temperature operation of a silicon qubit
Source: Sci Rep. 2019 Jan 24;9:469. doi: 10.1038/s41598-018-36476-z (PMC6346053; doi:10.1038/s41598-018-36476-z)
Supplement: Supplementary file 1 — Supplementary Information [file 41598_2018_36476_MOESM1_ESM.pdf]

# Supplementary Information for High-temperature operation of a silicon qubit

Keiji Ono, Takahiro Mori, and Satoshi Moriyama

## Fabrication of TFETs

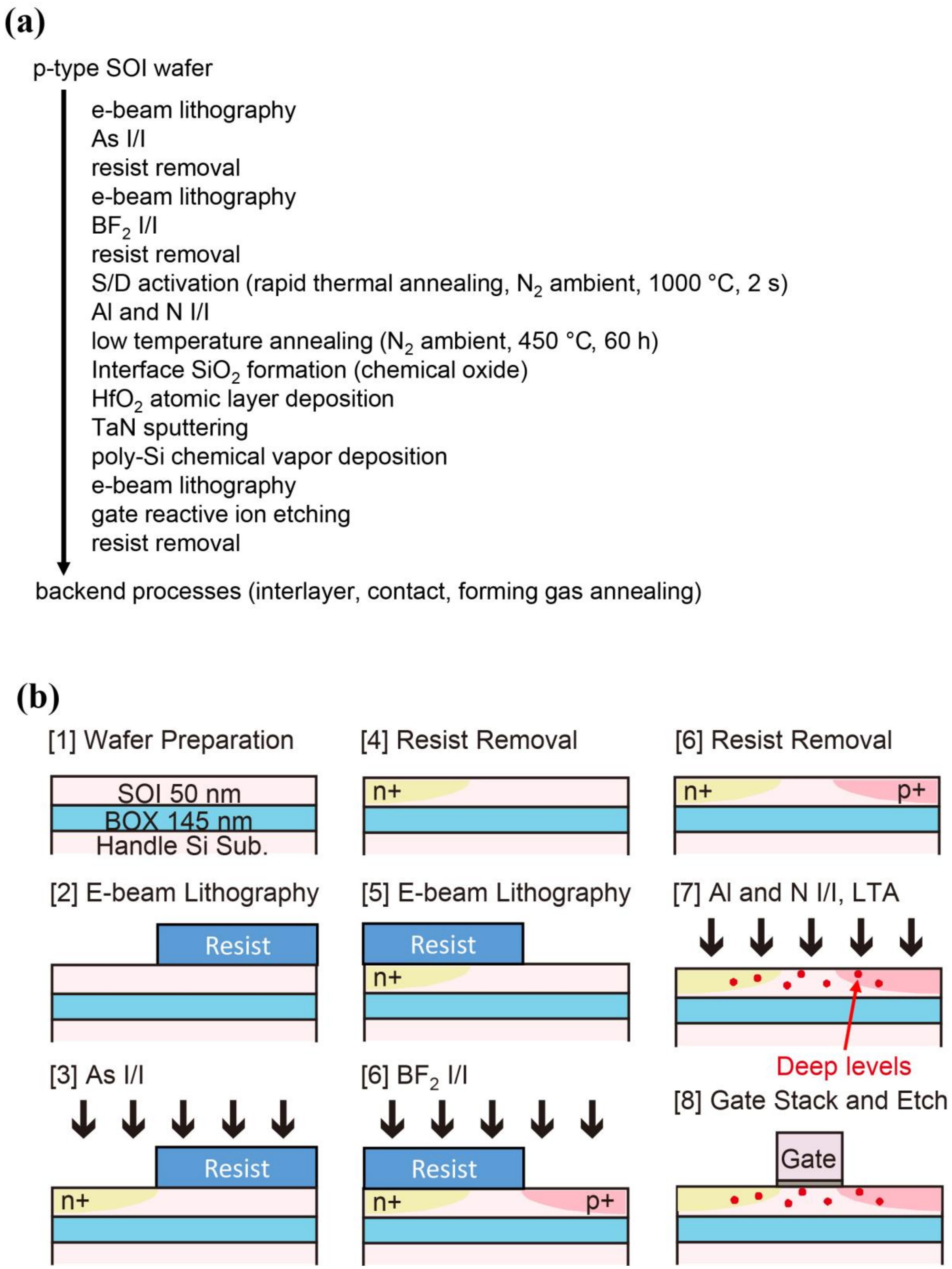


**Fig. S1. Fabrication of TFETs.** (**a**) Device fabrication process. (**b**) Schematic cross-sections of some of the steps in the process.

TFETs are fabricated via a process similar to that used to produce MOSFETs, which are the bases of conventional LSIs. TFETs are gated p-i-n diodes, in which the tunnelling current flowing through a pn junction is regulated at the source-side edge of the gate using electrostatic gate control. For example, in P-type TFETs, the source is n-type, whereas the drain is p-type. It should be noted that the source and drain definitions used in this paper are the same as those in the conventional P-type TFET case, although quantum transport of TFETs is discussed in this report.

We fabricated the TFETs on SOI wafers (Fig. S1). The SOI thickness was approximately 50 nm, and the BOX thickness was 145 nm. The top wafer surface was (100), and the current flow direction was <110>. First, I/I was employed to produce the source and drain. The source was formed by BF2 I/I with energy of 5 keV and a dose of $2 \times 10^{15}$ cm$^{-2}$. The drain was formed by As I/I with the same energy and dose. Rapid thermal annealing was performed at 1000 °C for 1 s to activate the source and drain. Then, we introduced isoelectronic traps (IETs) into the active region by I/I of Al and N with energy of 15 keV and a dose of $2 \times 10^{13}$ cm$^{-2}$. Low-temperature annealing was conducted at 450 °C for 60 h to activate the Al–N IETs. Following the introduction of the IETs, the deep levels we utilised in this work were formed in the channel. Finally, the gate was produced using high-k/metal gate technology. The interfacial $SiO_2$ was chemically formed with a thickness of approximately 1 nm, and $HfO_2$ (2.4 nm) was deposited onto it by performing atomic layer deposition at 250 °C. A TaN gate was formed by sputtering with a thickness of 10 nm, and a 50-nm doped-poly-Si cap was deposited by conducting chemical vapour deposition. This gate structure is also referred to as a metal-inserted-poly-Si gate. The equivalent oxide thickness of the gate insulator was estimated to be approximately 1.5 nm based on capacitance-voltage measurements. The IET technology utilised in this work has been proposed to improve the active performance of Si-based TFETs by enhancing the ON current. It should also be noted that the Al–N IET dose was 10 times higher than that in previous reports on conventional TFETs[20, 21, 22].

In this study, we fabricated the TFETs using the 100-mm fabrication facilities at AIST. Many kinds of TFETs, such as ones with different gate lengths and widths, were simultaneously fabricated on each wafer. The long-channel TFETs with gate lengths longer than 100 nm were successfully operated as conventional TFETs. All of the transistors were operational; also, their variations were suppressed effectively[46]. On the other hand, the short-channel TFETs with gate lengths shorter than 90 nm exhibited a short-channel effect in which the OFF current increased because the drain bias more strongly affected the channel potential. The short-channel TFETs operated as quantum transport devices, as reported in this manuscript.

**Differential conductance maps of TFETs (conventional short-channel and Al–N-implanted long-channel)**

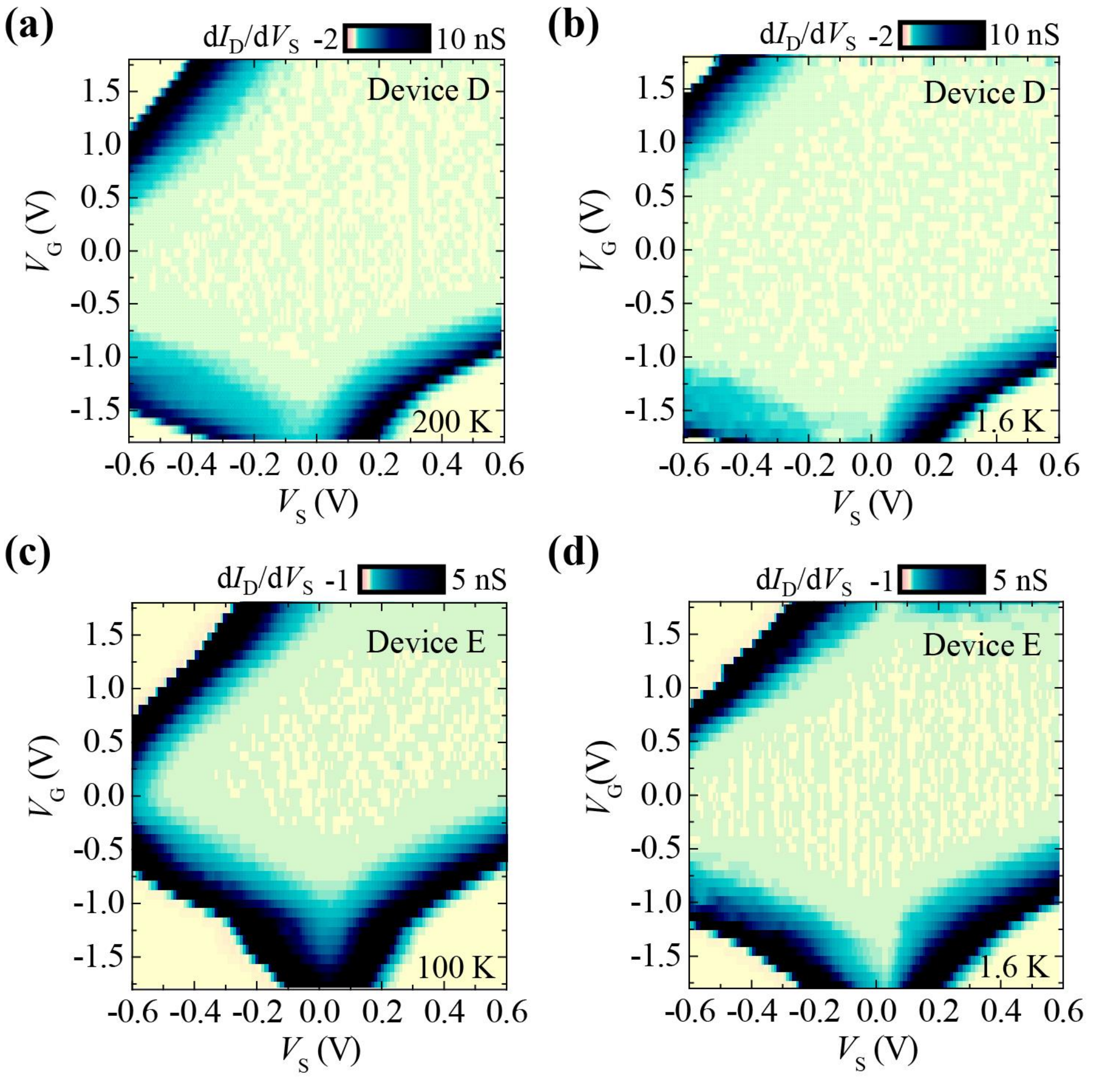


**Fig. S2. Differential conductance maps of TFETs.** (**a**), (**b**) d$I_D$/d$V_S$ intensity maps obtained at 200 K and 1.6 K, respectively, for device D, which was the TFET without Al–N implantation and with a channel length of 60 nm. (**c**), (**d**) d$I_D$/d$V_S$ intensity maps obtained at 100 K and 1.6 K, respectively, for device E, which was the Al–N-implanted TFET with a channel length of 100 nm.

The TFETs without Al–N implantation did not produce Coulomb diamonds, although they had short channels, as shown in Fig. S2(a) and (b) for the device with a channel length of 60 nm as an example. The Al–N-implanted TFETs with long channels also did not produce Coulomb diamonds, as shown in Fig. S2(c) and (d). The increase in d$I_D$/d$V_S$ in all four corners of the maps are in accordance with those observed in conventional TFET operation. The increase in the lower-right corners originated from band-to-band tunnelling between the channel (in which holes were generated when negative gate bias was present) and n-type source. Band-to-band tunnelling is also observable in the upper-right corner of Fig. S2(d), where tunnelling occurs between the channel (in which electrons were generated) and the p-type drain. The increase in the upper- and lower-left corners originated from the diffusion current, which corresponds to the forward-bias characteristics of conventional pn diodes. These features are mostly independent of temperature.

## Temperature dependence and negative Coulomb staircase of device A

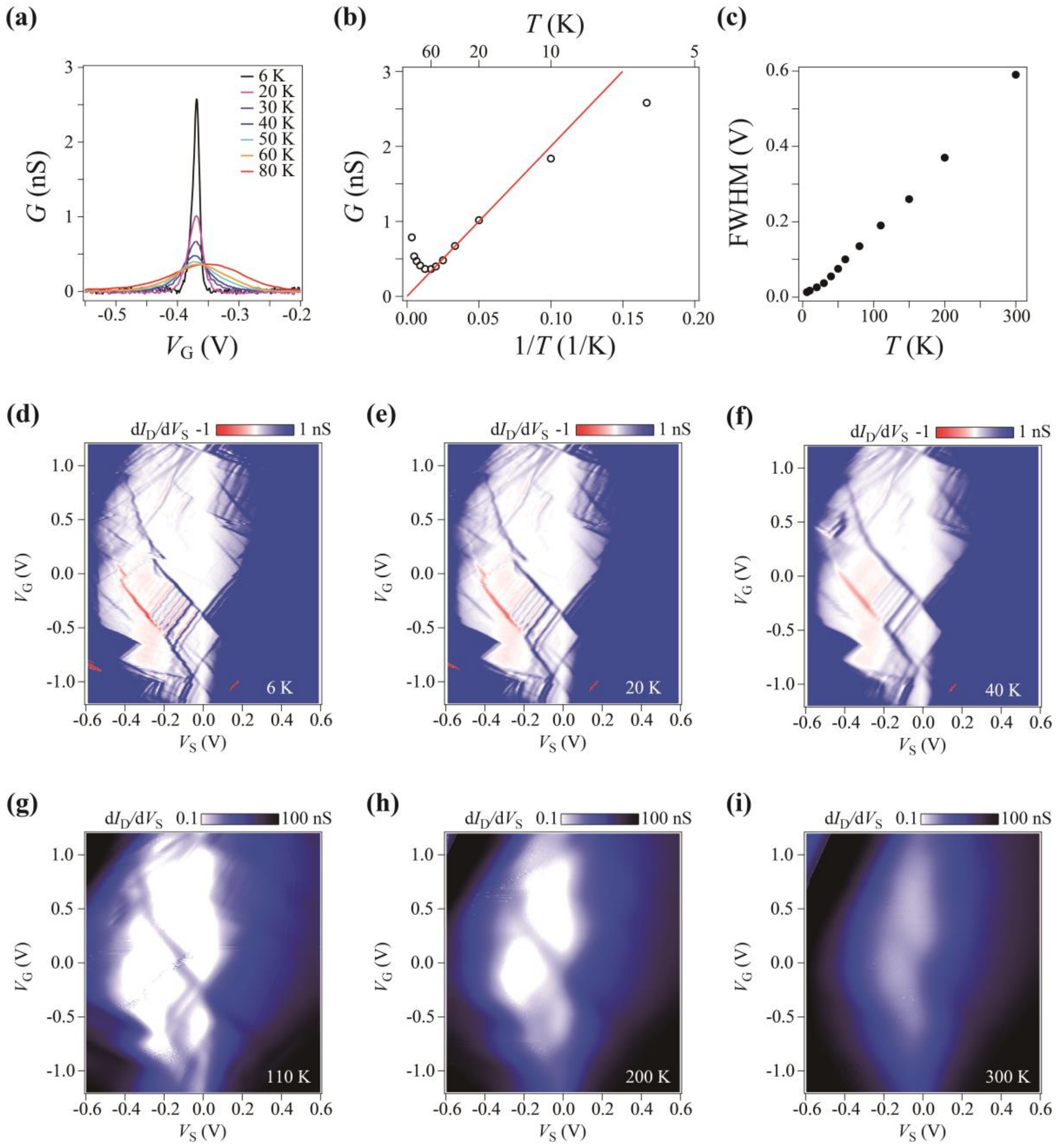


**Fig. S3. Temperature dependence and negative Coulomb staircase of device A.** (**a**) Detailed temperature dependence of the conductance peak in Fig. 2(c) for the lower temperature range. The peak was observable at temperatures up to 300 K. (**b, c**) Temperature dependences of the peak height (b) and width (c) of the conductance peak. (**d, f**) Linear-scale $dI_D/dV_S$ intensity maps measured at 6 K (**d**), 20 K (**e**), and 40 K (**f**). (**g, f**) Log-scale $dI_D/dV_S$ intensity maps measured at 110 K (**g**), 200 K (**h**), and 300 K (**i**).

Figure S3(a–c) present the detailed temperature dependence of the conductance peak in device A, which was observable at temperatures up to 300 K. As indicated by the red line in Fig. S3(b), for 20 K $\leq T <$ 60 K, resonant tunnelling through the dot is dominant, and the conductance peak shapes agree closely with those obtained from the theoretical expression for the quantum Coulomb blockade[47], where the peak height is proportional to $1/T$. At lower temperatures ($T <$ 20 K), the conductance peak height appears to approach saturation. For 60 K $\leq T <$ 110 K, the

conductance peak height is independent of temperature. The transition from quantum to classical Coulomb blockade occurs in this range. At temperatures greater than 110 K, the conductance peak height increases. This is not in accordance with classical Coulomb blockade theory, probably because of superposition of the tails of neighbouring peaks, current leakage and generation, and parallel conduction paths in the channel. The transition temperature around 60 K is in agreement with the level spacing between the first excited and ground states, $\Delta E$ = 20 meV, as is observable in Fig. S3(d–f). The conductance peak width corresponds to ~4 $k_B T$ for a quantum Coulomb blockade; in this case, 4 $k_B T$ is nearly 20 meV when $T$ = 60 K. The FWHM of the conductance peak monotonically increases with increasing temperature, mostly following the theory. The charging energy of 0.1 eV estimated from the temperature dependence of the Coulomb peak width is consistent with that estimated from the Coulomb diamonds.

Figure S3(d–f) depict the linear-scale intensity maps of $dI_D/dV_S$ at low temperatures. Negative differential conductance is observable in the red regions with $V_S$ ranging approximately from −0.2 V to −0.4 V and $V_G$ approximately from 0 V to −0.6 V. The thick red lines slanting down to the right correspond to the negative staircase in Fig. 2(b). The thin red lines slanting down to the left are along the edges of the Coulomb diamonds and parallel to the excited states. In previous studies, it was theoretically predicted that negative Coulomb staircases should be observed in single-molecule devices in which single-electron transport is coupled to the vibration mode of the molecule[48, 49]. Therefore, it is suggested that the quantum dots in our devices were atomically small, so molecule-like vibration modes existed. We suppose that the vibrations originated from local phonon modes of the deep impurities.


[47] Beenakker, C. W. J. Theory of Coulomb-blockade oscillations in the conductance of a quantum dot. *Phys. Rev. B* **44**, 1646 (1991).

[48] Koch, J. & von Oppen, F. Franck–Condon blockade and giant Fano factors in transport through single molecules. *Phys. Rev. Lett.* **94**, 206804 (2005).

[49] Härtle, R. & Thoss, M. Resonant electron transport in single-molecule junctions: Vibrational excitation, rectification, negative differential resistance, and local cooling. *Phys. Rev. B* **83**, 115414 (2011).

**Temperature-dependent characteristics of device B**

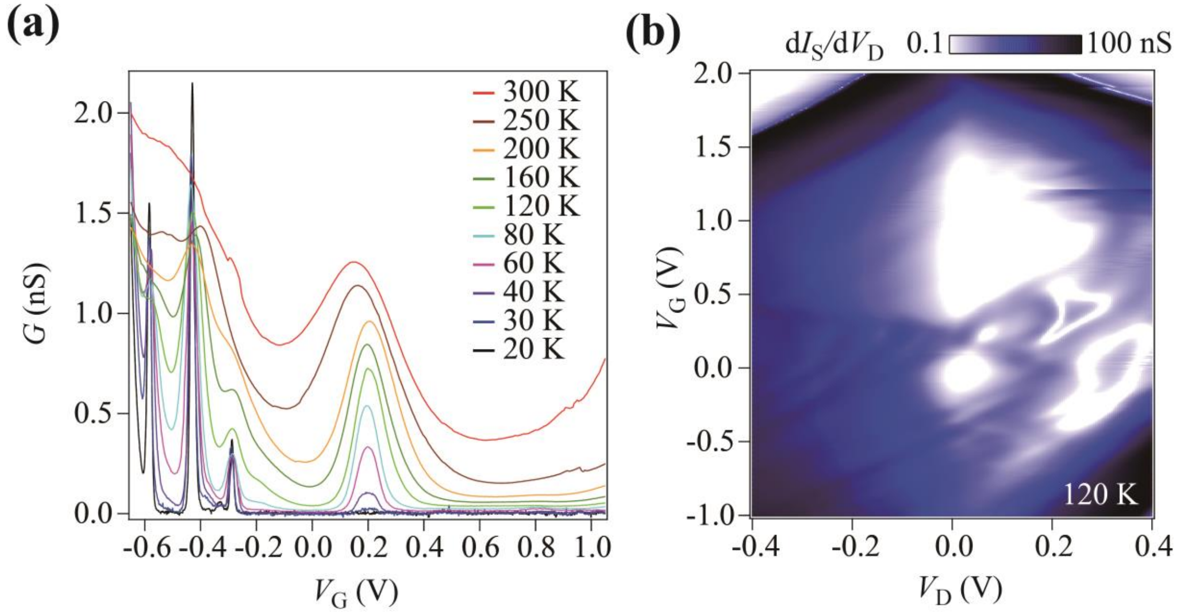


**Fig. S4. Temperature-dependent characteristics of device B.** (**a**) Temperature dependence of the zero-bias conductance in device B. (**b**) Log-scale d$I_S$/d$V_D$ intensity map measured at 120 K.

Figure S4(A) shows the temperature dependence of the zero-bias conductance in device B. The peak at $V_G$ ~ 0.2 V is observable at temperatures above 40 K; however, it is suppressed below 30 K. The peak position is in the double-quantum-dot transport region (Fig. 3(a)). Therefore, the suppression at low temperatures is due to the double dot formation, and the peak appears at high temperatures due to the transition from double to single dot because the weakly confined dot vanished. The peak is observable up to 300 K, which indicates that one of the dots had strong confinement energy originating from a deep level. Figure S4(b) shows the log-scale d$I_S$/d$V_D$ intensity map measured at 120 K. Large, closed Coulomb diamonds are observable, with maximum widths at $V_G$ = 0 V and 0.9 V corresponding to large single-electron charging energies of 0.2 eV and 0.3 eV, respectively. This strongly confined single dot behaviour can be described as discussed above.

## Landau-Zener-Stuckelberg-Majorana interference in device B

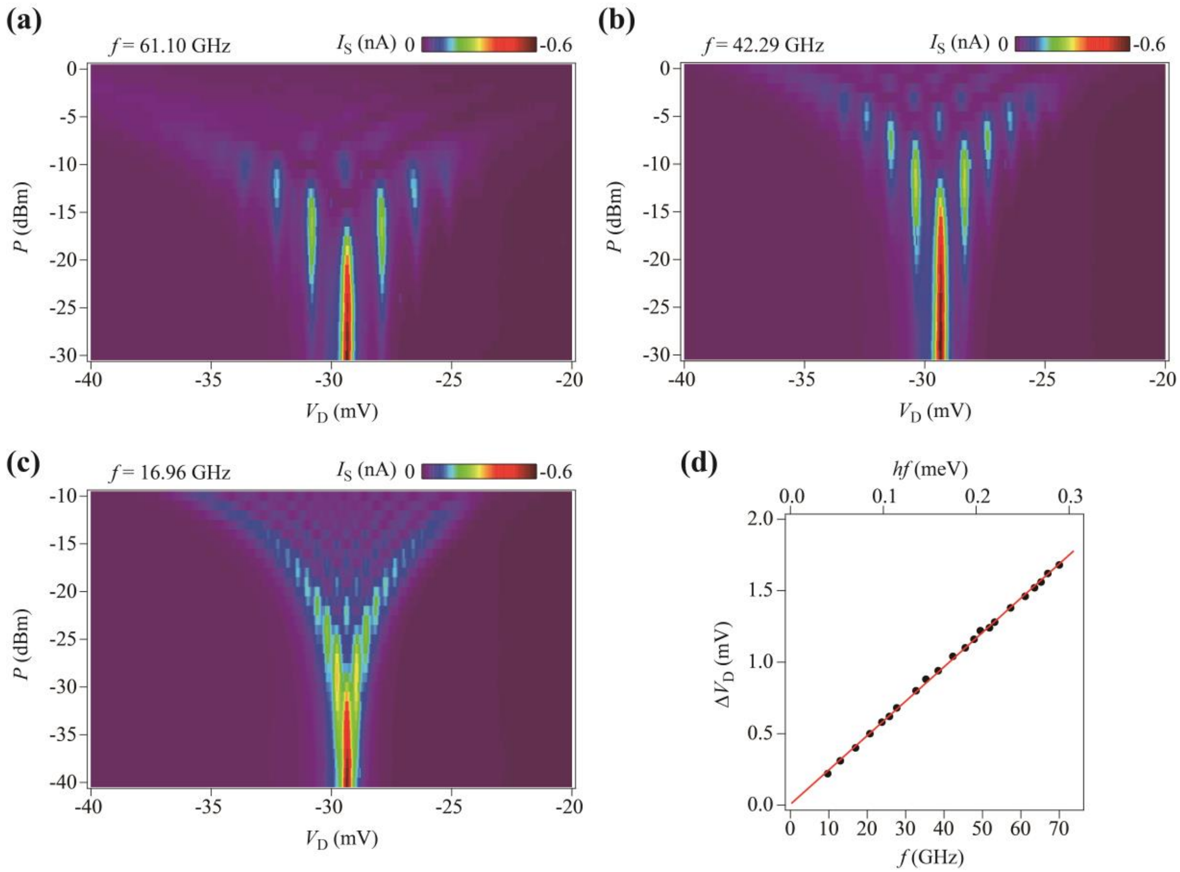


**Fig. S5. Landau-Zener-Stuckelberg-Majorana interference in device B.** (**a–c**) Colour intensity maps of $I_S$ near the sharp peak at $V_D = -0.03$ V (Fig. 3(b)) measured at 1.5 K as functions of $V_D$ and $P$ at $V_G = 0.255$ V with constant microwave frequencies of 61.10 GHz (a), 42.29 GHz (b), and 16.96 GHz (c). (**d**) Voltage difference $\Delta V_D$ between the first satellite peaks and main peak as a function of the applied microwave frequency.

Figure S5(a–c) present the dependence of the sharp $I_S$ peak at $V_D = -0.03$ V (Fig. 3(b)) on $P$. We observed Landau-Zener-Stuckelberg-Majorana interference at frequencies ranging from 10 GHz to 70 GHz. The dependence of the peak height on $P$ agrees with the theoretical square Bessel function expression[35]. Landau-Zener-Stuckelberg-Majorana interference is observable even if the energy of one photon $hf$ is smaller than the measurement temperature (1.5 K). $\Delta V_D$ depends linearly on the frequency between 10 GHz and 70 GHz, as shown in Fig. S5(d). Using the slope of the line, we estimated the conversion factor between the drain voltage and energy in the dot as 0.17 meV/mV (17%). Using this factor, the $I_S$ peak width was converted into energy of 0.06 meV, which is less than that corresponding to the measurement temperature (0.12 meV, corresponding to 1.5 K). This relationship indicates that the electron transport is limited by the lifetime of the single-particle states in the quantum dots and is independent of temperature, and provides evidence of resonant tunnelling in the series-coupled double quantum dot.

## ESR in device B

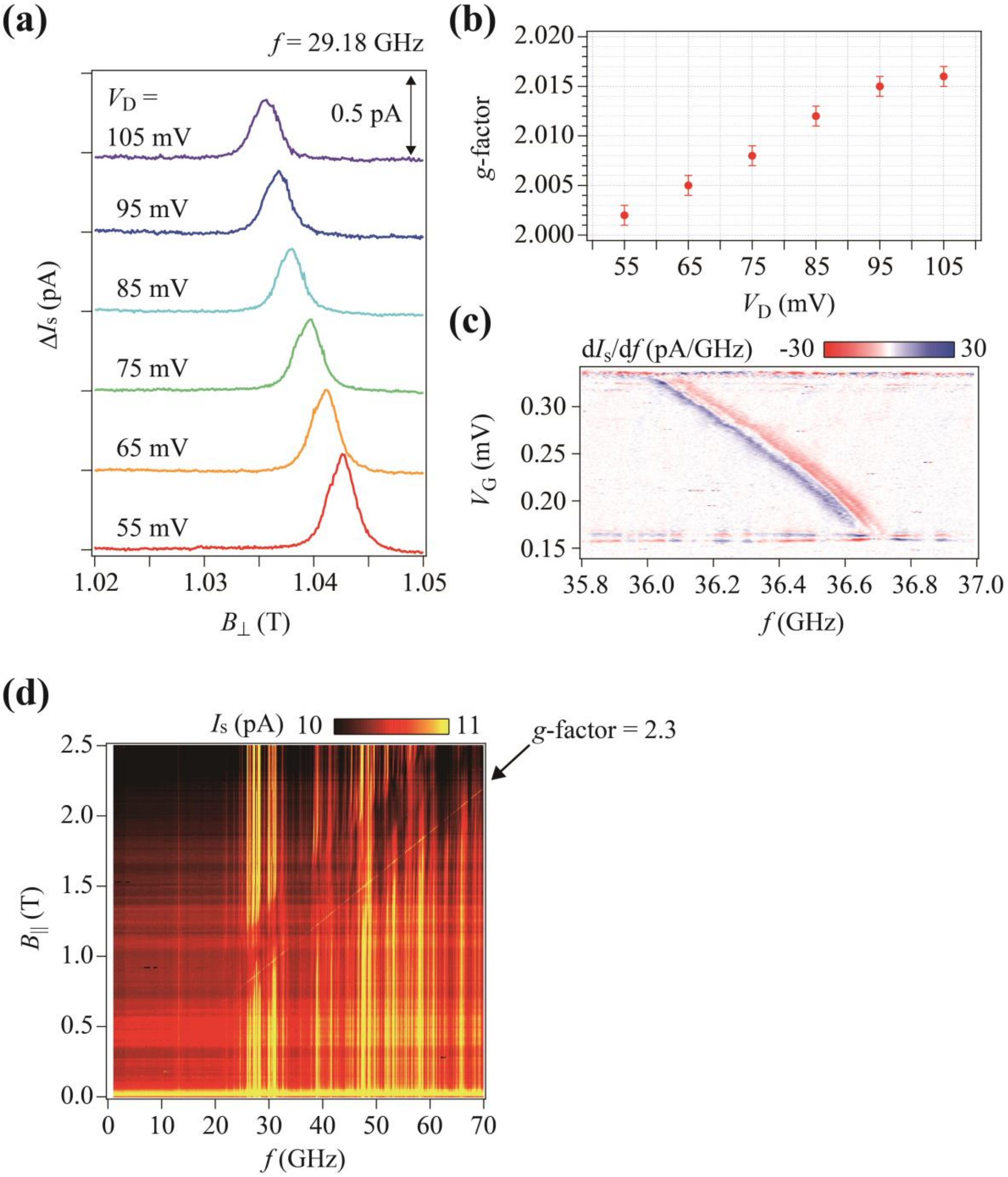


**Fig. S6.** (**a**) $V_D$ dependence of the ESR peak as a function of $B_\perp$ with $V_G$ = 0.253 V, $f$ = 29.18 GHz, and $P$ = 3 dBm. Each curve is artificially shifted by 0.5 pA from the one below it for clarity. (**b**) $V_D$ dependence of the $g$-factor obtained from Fig. S6(a) and similar measurements with several frequencies and magnetic fields. We performed measurements with various $B_\perp$ and confirmed that the peak position vs. $B_\perp$ line passed through the origin. (**c**) $V_G$ dependence of the ESR peak ($dI_S/df$) as a function of microwave frequency with $P$ = 3 dBm, $V_D$ = 0.075 V, and $B_\perp$ = 1.3 T. For 0.16 V < $V_G$ < 0.33 V, the ESR response is observable because a spin blockade occurred in this range (see also Fig. 3(a)). (**d**) $I_S$ intensity of device B at ($V_D$, $V_G$) = (0.055 V, 0.14 V) as a function of $B_{||}$ and microwave frequency with $P$ = 3 dBm and different cooldowns, measured at 1.5 K.

Figure S6(a) shows the $V_D$ dependence of the ESR peak as a function of $B_\perp$ in the spin blockade transport region (see also Fig. 3(a)). We performed similar measurements using several frequencies and magnetic fields, and the *g*-factors obtained from the dependence of the ESR peak position on $B_\perp$ for each $V_D$ are summarised in Fig. S6(b). Figure S6(c) depicts the $V_G$ dependence of $dI_S/df$ in the spin blockade transport region (see Fig. 2(a)). The change in the peak position is attributed to the change in the *g*-factor from 1.98 to 2.01. Specifically, in the spin blockade region, which was the area enclosed by the dotted line in Fig. 3(a), the *g*-factor changed by approximately 2%.

Figure S6(d) presents the ESR spectra of device B obtained with different $B_\parallel$ and different cooldowns. After several thermal cycles in which the temperature varied from 300 K to 1.5 K, the structure of the charge stability diagram hardly differs from that in Fig. 3(a), but the diagram is shifted towards negative gate voltages by about 0.1 V, probably because of the generation of fixed charge in the gate oxide due to the thermal cycling. Considering the voltage shift, we performed the same measurements as those used to obtain Fig. 3(c), except with a different magnetic field direction, and obtained a *g*-factor of 2.3.

## Coulomb diamond and ESR spectra of device C

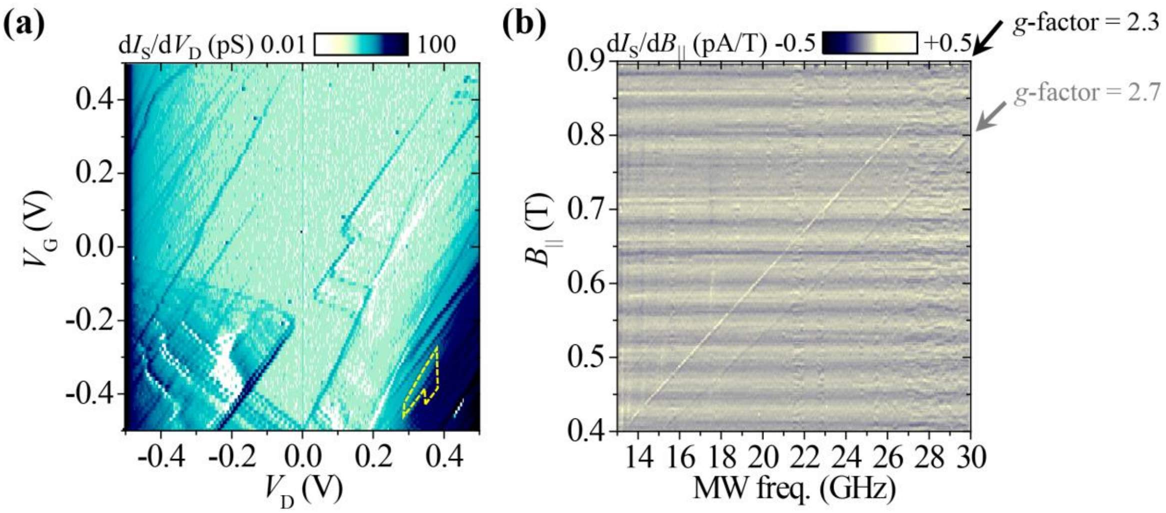


**Fig. S7. Coulomb diamond and ESR spectra of device C.** (**a**) d$I_S$/d$V_D$ intensity map obtained at 1.5 K, in which the spin blockade area is enclosed by a yellow dotted line near the lower-right corner. (**b**) d$I_D$/d$B_{||}$ intensity map measured at ($V_D$, $V_G$) = (0.33 V, −0.36 V) and 1.5 K as a function of $B_{||}$ and microwave frequency.

The Coulomb diamond and ESR spectra of device C that were measured at 1.5 K are presented in Fig. S7. The intensity of d$I_S$/d$V_D$ is weaker than it was for device B because the channel length of device C was longer than that of device B, which resulted in weaker tunnel coupling in device C. The open (unclosed) diamond at $V_G$ = −0.15 V suggests that multiple dots were formed in the channel. We measured the ESR responses for every other ($V_D$, $V_G$) set in 10 mV intervals in Fig. S7(a). ESR response similar to that in Fig. 4(a) occurred in the area enclosed by the yellow dotted line in Fig. S7(a). This area is smaller than the corresponding area for device B, and its shape is different from the conventional one, which suggests that complicated parallel conduction paths existed in the channel.

Figure S7(b) shows the ESR spectra measured in the spin blockade region. Two ESR lines with $g$-factors of 2.3 and 2.7 are observable. The peak intensity of the ESR with the $g$-factor of 2.7 is weak, so it is barely recognizable in the d$I_D$/d$B_{||}$ map. Thus, pulsed ESR could not be conducted with that ESR peak.

## Summary of other short-channel Al–N-implanted TFETs

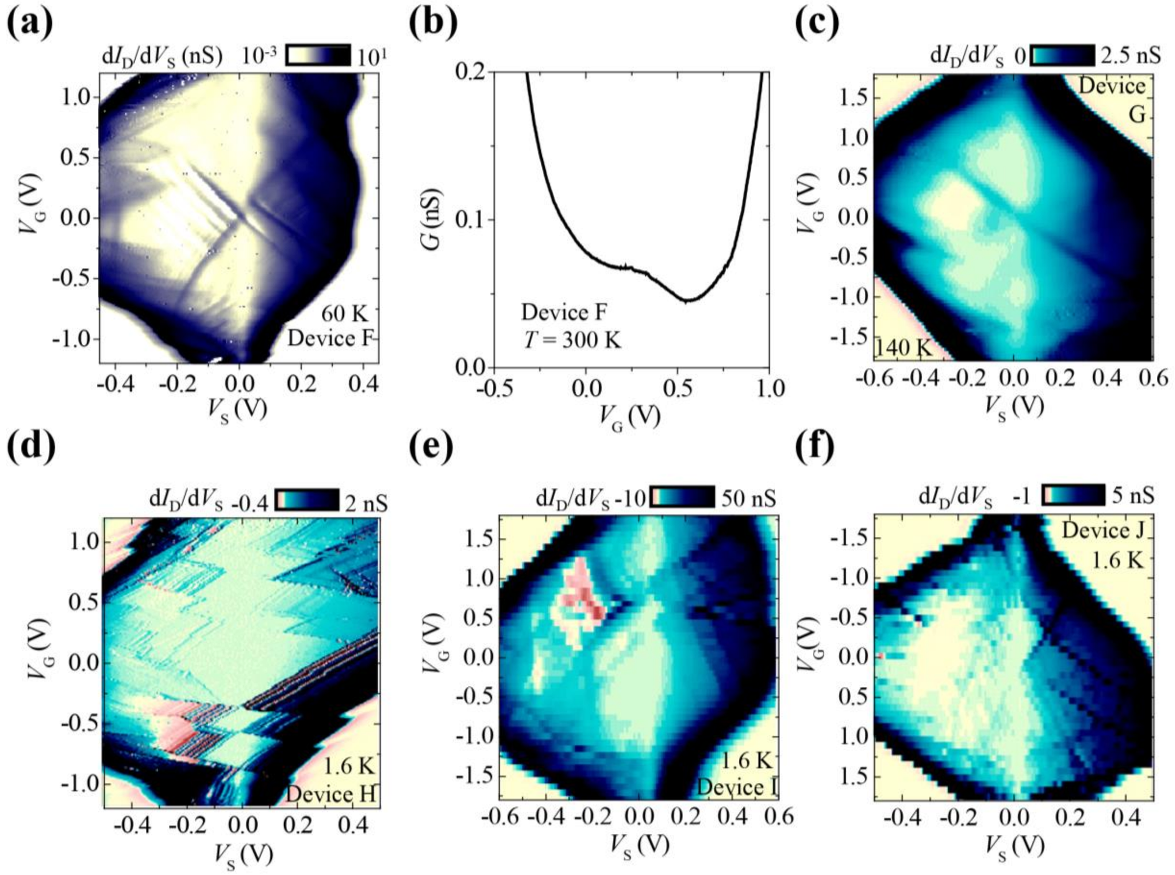


**Fig. S8. Characteristics of five Al–N-implanted short-channel TFETs.** (**a**) $dI_D/dV_S$ intensity map measured at 60 K for device F, which had a channel length of 70 nm. (**b**) $G$–$V_G$ curve measured at 300 K for device F. (**c**) $dI_D/dV_S$ intensity map measured at 140 K for device G, which had a channel length of 70 nm. (**d–f**) $dI_D/dV_S$ intensity maps measured at 1.6 K for device H, which had a channel length of 70 nm (**d**); device I, which had a channel length of 60 nm (**e**); and device J, which had a channel length of 60 nm (**f**).

As discussed in the main text, we characterised 41 devices with channel lengths of 60, 70, and 80 nm. Among them, 37 devices, all of which had high single-electron charging energies, exhibited single- or multiple-quantum-dot transport. Furthermore, three devices (devices A, B, and F) exhibited single-electron transport at room temperature. The characteristics of device F are depicted in Figs. S8(a) and S8(b). Device G (whose characteristics are illustrated in Fig. S8(c)) operated at temperatures up to 140 K, although the charging energy was as high as those of the three aforementioned devices were. The characteristics of three additional devices are provided in Fig. S8(d–f) as examples of multiple-dot-like transport at 1.6 K.
